# Investigation of Hafnium Thin Films for Design of TES Microcalorimeters

**DOI:** 10.3390/ma17010222

**Published:** 2023-12-30

**Authors:** Victoria Yu. Safonova, Anna V. Gordeeva, Anton V. Blagodatkin, Dmitry A. Pimanov, Anton A. Yablokov, Olga L. Ermolaeva, Andrey L. Pankratov

**Affiliations:** 1 Superconducting Nanoelectronics Laboratory, Nizhny Novgorod State Technical University n.a. R.E. Alekseev, Minin Street, 24, 603155 Nizhny Novgorod, Russiaa.gordeeva@nntu.ru (A.V.G.);; 2 Institute for Physics of Microstructures of the Russian Academy of Sciences, Academicheskaya Street, 7, 603950 Nizhny Novgorod, Russia

**Keywords:** hafnium, thin films, TES, microcalorimeters, cryogenic measurements, superconductivity, critical temperature, proximity effect

## Abstract

Hafnium is a superconductor with a transition temperature slightly above 100 mK. This makes it attractive for such applications as microcalorimeters with high energy resolution. We report the superconducting properties of Hf films of thicknesses ranging from 60 to 115 nm, deposited on Si and Al_2_O_3_ substrates by electron beam evaporation. Besides that, we fabricated and measured combinations of hafnium with thin layers of normal metals, decreasing the critical temperature by the proximity effect. The critical temperature of the studied films varied from 56 to 302 mK. We have observed a significant change in the critical temperature of some films over time, which we propose to prevent by covering hafnium films with a thin layer of titanium.

## 1. Introduction

Hafnium is a transition metal known for its unique characteristics at low temperatures. This material is a promising candidate for various applications, including X-ray spectrometry [1], single photon counters in microwave [2,3], far infrared [4], and optical range [5]. Besides that, hafnium thin films can potentially be used for the fabrication of a microcalorimeter based on a transition-edge sensor (TES) due to the possibility of acquiring the transition temperature below 100 mK. There are such challenging applications as the detection of individual He atoms in superfluid helium for the neutrino magnetic moment search [6] and the dark matter search [7]. They require a material for a TES that has a critical temperature below 100 mK and a narrow width of the superconducting transition. Moreover, for a successful operation, the properties of a thin film material must be stable over time.

The TES sensitivity depends on the volume of the sensor. The higher the critical temperature *T*_C_ of the base material, the thicker the layer of a normal metal is needed to suppress *T*_C_ to the required values by the proximity effect. In this regard, the latest TES generations for operating temperatures of 30 to 50 mK are based on such materials as tungsten and iridium [8,9,10]. The values of their critical temperatures, 15 mK and 112 mK, respectively, are the closest to the required ones [11,12,13,14]. The critical temperature of bulk hafnium is comparable with that of iridium [1,14,15] and can be further suppressed using normal metal layers, such as silver or gold.

So far, hafnium has been poorly studied for TES applications. Works that investigate the properties of Hf films show a broad spread of the critical temperature, from 50 to 495 mK [5,14,15]. The latter depends on the substrate material, the deposition method, and presumably other factors specific to each case. For example, in [15], they give the dependence of *T*_C_ on the argon pressure in the chamber, and the critical temperature varies from 250 to 450 mK. At the same time, in [1], for bulk hafnium, the critical temperature was 137–205 mK with a transition width of 2 mK. The superconducting transition temperature of hafnium thin films was reported to be about 200 mK for a TES detector of 25 × 25 μm in size with a film thickness of 30 nm [13].

Hafnium has some advantages over other materials that have critical temperatures around 100 mK. The iridium critical temperature can also be suppressed by the proximity effect [9,12,16,17]. But iridium is more expensive compared to hafnium, and the iridium technology is more complicated since it requires substrate heating and magnetron sputtering [8,10]. While tungsten has a critical temperature close to 15 mK [18], it has its drawbacks, such as several crystal modifications that can turn into each other during thermal cycles [13]. In contrast, hafnium has a hexagonal, close-packed crystallographic structure that is not as susceptible to temperature loads as tungsten with its A15 crystallographic structure [18].

And it should be noted that under very high pressure the critical temperature of hafnium rises up to about 8 K [19]. This is due to the *ω*-*β* phase transition, which happens at the pressure between 60 and 64 Gpa.

The purpose of this work is to investigate the properties of hafnium thin films and their combinations with normal metal films in relation to the fabrication of a TES microcalorimeter. During measurements, we have observed the aging effects of single- and double-layer film properties. These aging effects and possible stabilization of film properties are discussed in the last section.

## 2. The Measurement Setup and the Sample Holder

The measurements were carried out in a standard dilution cryostat Triton 200 from Oxford Instruments (Abingdon, UK), with a minimal temperature of 10 mK. Film samples were mounted on the lower plate of the cryostat under the dilution chamber.

We made a brass holder designed to accommodate 10 samples. The holder has a thermal screen, preventing the background radiation effect from hotter fridge plates. Due to the clamping contacts, the holder allows mounting films with sizes ranging from 4 × 4 mm to 12 × 20 mm. The distance between the contacts is 2.54 or 5.08 mm, depending on the film size.

These clamping contacts have several advantages:

- A good thermal contact of the measured film with the metal base of the holder;
- Easy sample changing;
- Ability to measure films of non-standard size and irregular shape.

**Figure 1:**
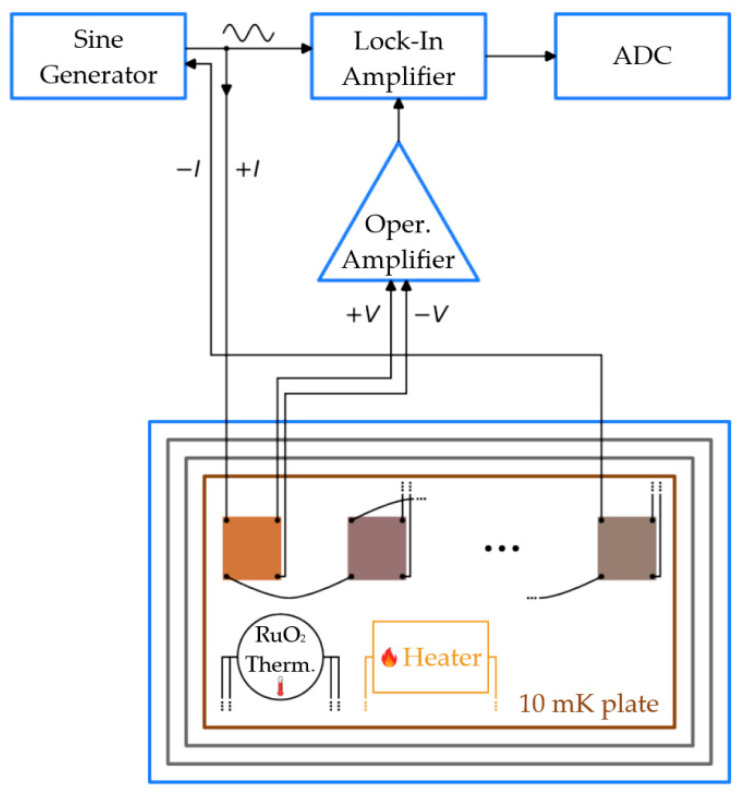
The measurement setup.

To set the current, only two contacts are used because all samples can be connected in series. This leaves more contacts for voltage measurements (the current and voltage supply diagram is shown in Figure 1).

The measurement setup to conduct the current-voltage (IV) characteristic measurements has been previously used for investigations of highly sensitive receivers, such as Cold-Electron Bolometers [20] and microwave Single Photon Counters [21]. It consists of a current source made of a pair of INA105 operational amplifiers (op-amps) and a couple of bias resistors. The latter were used to convert the voltage source into a current source. The magnitude of the current depended on the voltage applied to the inputs of the op-amps. So, the samples were studied in a current-bias mode. The voltage across a sample came back to a couple of AD745 op-amps to be multiplied by the factor of 100. The voltage applied to the INA105 inputs came from a National Instruments PXI-6733 board (Austin, TX, USA), and the voltage acquired from the AD745 outputs went to an NI PXI-4472B board. The IV curves were measured slowly, taking about 10 s to ramp the current up and another 10 s to ramp the current down. Figure 1 depicts the scheme of the setup for measuring resistance versus temperature *R*(*T*) to characterize the superconducting transition. This time, the voltage applied to the INA105 inputs came from the sine generator of the SR-830 lock-in amplifier. Then, the outputs of the AD745 op-amps came into the input of the lock-in amplifier. The latter sensed the signal at the probe frequency set to 315 Hz. The in-phase and quadrature components of the response were digitized using the built-in analog-to-digital converter (ADC).

Dividing the voltage by the current and considering the gain of the lock-in amplifier, we calculated the film resistance. We slowly changed the temperature of the plate to get the temperature dependence of the required film resistance *R*(*T*).

The temperature was measured with a commercial RuO_2_ thermometer supplied with the cryostat, calibrated with a Magnicon SQUID thermometer (Berlin, Germany). One temperature measurement with this thermometer takes at least 15 s, depending on the settings. The films were heated using a built-in heater on the 10 mK plate of the cryostat.

## 3. Measurement Results

We investigated hafnium films with different thicknesses in the range from 60 to 115 nm, deposited on substrates by electron beam evaporation. We studied how thin layers of silver and gold affect the critical temperature of superconducting Hf films.

All the films investigated here were deposited using electron beam evaporation, since amorphous hafnium films with a smooth surface can be obtained by this method [22]. Moreover, hafnium can be deposited by magnetron sputtering. However, the sputtered films normally have higher critical temperatures, which contradicts our goal.

We have tested two different hafnium sources with purity levels of 99.95% and 99.9%. These sources led to superconducting films in most cases. Below, the results of measurements are presented for films made from the most pure source (99.95%, named Hf-1 for convenience) and from another source (99.9%, denoted as Hf-2). In general, we did not find any noticeable difference in superconducting properties between the films of Hf-1 and Hf-2.

### 3.1. Resistivity Measurements of Hafnium Films

The differential resistance was obtained from the slope in the current-voltage characteristics (IV) of hafnium film samples. When the differential resistance became constant, we assume that the film had reached its normal state. As the IV curve of a normal metal crosses zero, we consider the final differential resistance to be equal to the full one. We used the latter to calculate the resistivity ρ. The following data presents the resistivity for each sample (Table 1). For the resistivity calculations, we should consider the deviations of film thickness and resistance at 300 K and at 20 mK. The thickness deviation was a few percent (taking into account a film roughness about 1 nm), and the error of the resistance measurement was about 5% at 300 K and close to 1% at 100 mK. So, the measurement error of resistivity should not exceed 7% at 300 K and 5% at 20 mK.

At room temperature, the resistivity of Hf-1 films is closer to the standard value of the bulk resistivity of hafnium (4 × 10^−7^ Ω × m) than one of the films of Hf-2. At 20 mK, the Hf-2 resistivity becomes equal to the bulk value, while the Hf-1 values are one order of magnitude lower. For all measured hafnium films, the lower the temperature is, the lower the resistance is. This implies that there is little influence of the impurities on the electronic transport for both hafnium sources. Therefore, considering the temperature decrease in the resistivity and its convergence to the bulk value, we can expect that the critical temperature of the films will be comparable with the values of the critical temperature of bulk hafnium indicated in [1].

### 3.2. Film-by-Film Results for Old Hafnium

#### 3.2.1. 97 nm Hf-1 Film

**Figure 2:**
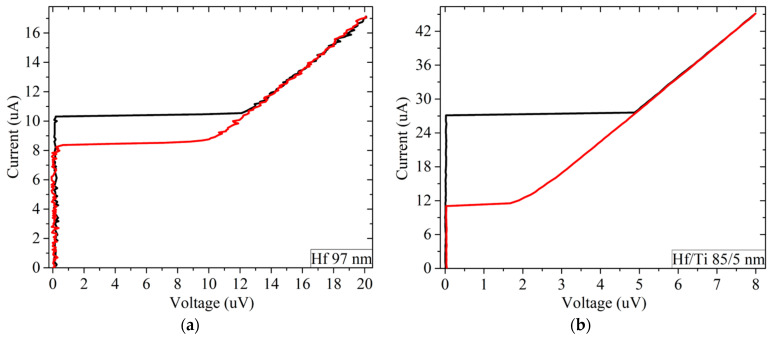
(**a**) The current–voltage characteristics of the Hf-1 97 nm; (**b**) The current-voltage characteristics of the Hf/Ti (Hf-2) 85/5 nm. Black line is for direct branch, red line is for reverse branch.

The 97-nm-thick hafnium film deposited on a Si substrate was measured at 15 mK and demonstrated the typical current-voltage characteristic of a film in the superconducting state (Figure 2a). The critical current is 10.4 μA, the normal resistance after the transition to the normal state is 1.1 Ω.

Figure 2 shows a typical current-voltage characteristic of the superconducting films that we measured. They differ only in normal resistance, critical current, and hysteresis. Here, the black curve is for the increasing current, and the red one is for the reverse branch.

When the current increases above 10.4 μA, the film jumps into the resistive state. Such a sharp transition suggests that the observed critical current is not the true critical current of a given film. Rather, the transition to the resistive state occurs due to the heating of the supply wires by the current, from which heat is also transferred to the film. The hysteresis between the branches is clearly visible, the return current is 8.2 μA. This indicates that the film has warmed up after the transition to the resistive state. The bulk Hf is a type I superconductor, but thin films are known to be a type II superconductor. According to Silsbee criterion the switching current has to be as high as 10 A, taking into account that the critical magnetic field of hafnium is 50 Gauss [23]. We conclude that the switching to the resistive state is not due to the magnetic field from the Silsbee criterion.

**Figure 3:**
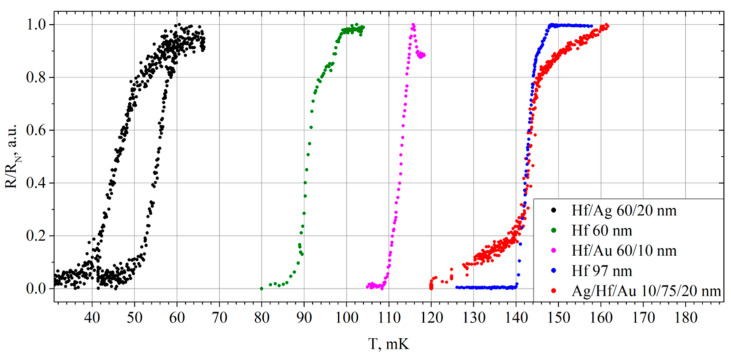
The dependence of the resistance on temperature of Hf-1 films relative to their normal resistances.

After measuring the IV curve, the critical temperature and the transition width were examined (Figure 3, the blue curve for the Hf 97 nm sample) based on the dependence of the film resistance on the phonon temperature. The bias current of 2 μA was supplied to the film, and the voltage was read using the Stanford Research SR-830 lock-in amplifier (Sunnyvale, CA, USA). The temperature of the cryostat plate was smoothly increased using built-in heaters. Further measurements of the temperature dependence of the resistance were carried out similarly.

The transition from the superconducting to the resistive state started at a temperature of 140 mK, the resistance reached a plateau at a temperature of 148 mK. Presently, the film has completely returned to its normal state with a constant resistance of 1.1 Ω. The transition turned out to be smooth and repeatable; only a small inflection was noticeable in the region of 145 mK. The main part of the transition from the superconducting to the normal state (from 0.2 to 0.8) turned out to be narrow, about 3 mK, indicating rather good film uniformity.

#### 3.2.2. 60 nm Hf-1 Film

The current-voltage characteristic of a 60-nm-thick hafnium film was measured at 20 mK in the cryostat. The critical current turned out to be 8 μA, the normal resistance at 20 mK was 1.8 Ω and the return current was 5 μA.

The green dots in Figure 3 show the dependence of the sample resistance on the temperature. The transition from the superconducting state to the normal one began at 87 mK and stopped changing at 99 mK, when the film completely turned into the normal state. The transition looks stepwise, inflections are noticeable in the region of 92 and 96 mK, but in the main section, the width of the transition is 5 mK.

#### 3.2.3. 60/10 nm Hf/Au (Hf-1) Film

A 60-nm-thick hafnium film with a 10-nm-thick layer of gold deposited on top without a vacuum break was fabricated and measured at the cryostat temperature of 17 mK. The critical current was 48 μA, the normal resistance was 0.98 Ω. There was a large thermal hysteresis of the IV curve, with the return current much smaller than the critical one, which indicated a strong overheating of the film. At the same time, such a strong hysteresis on the IV curve can be used to create microwave single-photon detectors based on Hf microbridges [2,3,14].

We performed the measurements of the critical temperature and the width of the superconducting transition at a bias current of 0.2 μA, which began at a temperature of 109 mK and finished at 117 mK (Figure 3, the magenta curve). The transition was relatively narrow and smooth, with 3 mK width. It should be noted that before the complete transition to the normal state, the film resistance in a certain temperature range turned out to be higher than the normal film resistance of 0.98 Ω. The most probable reason for this is the increase in electron temperature and a large amount of nonequilibrium quasiparticles due to a rather large bias current and improper shielding of the sample from background radiation. This was one of the first samples measured, and we fixed these shortcomings later with a modified sample holder with additional filters.

#### 3.2.4. 10/75/20 nm Ag/Hf/Au (Hf-1) Film

We deposited a three-layer film of 10 nm silver, 75 nm hafnium, and, finally, 20 nm gold as the top layer on a Si substrate. The critical current at a temperature of 20 mK was 22 μA, the normal resistance after the transition to the resistive state was 0.13 Ω. A small thermal hysteresis was seen and the return current decreased to 20 μA.

The critical temperature and the transition width were measured at the bias current of 5 μA. The transition from the superconducting state to the normal one began at a temperature of 142 mK and continued to 146 mK (Figure 3, the red curve). At this point, the film has completely switched to its normal state. Although the beginning and end of the transition process were smooth, the transition itself was quite narrow. In the linear section with a constant slope, it was 5 mK, which coincides with that of the 97-nm-thick pure hafnium film.

**Figure 4:**
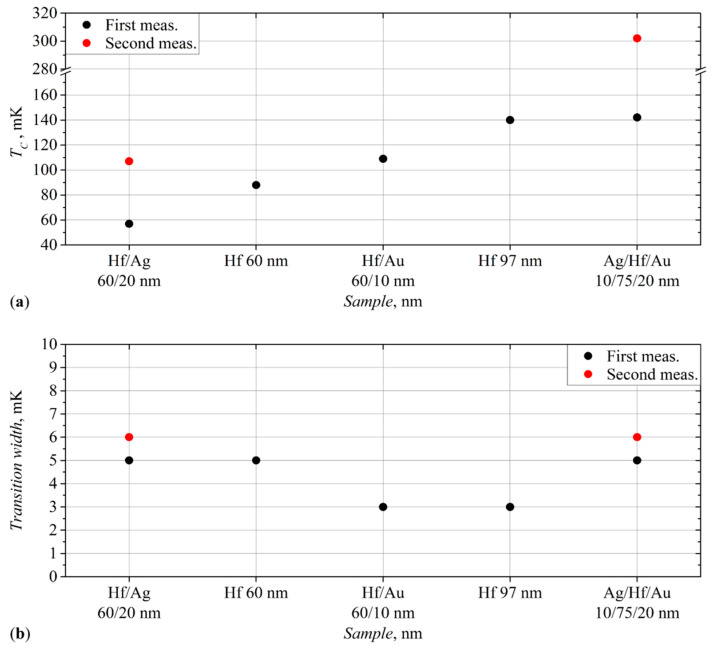
(**a**) The critical temperatures of the Hf-1 films in ascending order; (**b**) The transition widths of the Hf-1 films. The point size is bigger than measurement uncertainties.

Several months after the deposition, this film was measured again and showed the critical temperature rise to 302 mK (Figure 4a, the red dots). The width of the transition has changed slightly (Figure 4b, the red dots). This measurement is described in more detail in Section 3.5, related to the aging effects of the film properties.

One should note that the error of the used RuO_2_ thermometer in the range close to 100 mK is about 1%. The aging effects were confirmed by sequential measurements of fresh and old samples in the same holder during the same cooldown.

#### 3.2.5. 60/20 nm Hf/Ag (Hf-1) Film

A 60 nm hafnium film with a 20 nm silver layer on top was measured in the cryostat literally immediately after electron beam deposition. From its IV curve, the critical current was found to be about 21 μA, and the normal resistance to be 0.2 Ω. On the reverse branch of the IV characteristics, the return current was 11 μA due to film heating.

The critical temperature and transition width in the first measurement can be seen in Figure 3 (the black curve). During heating, the transition from the superconducting state started at 56 mK, and the film switched to the normal state at 64 mK. On cooling, the film resistance began to drop at 52 mK, and it completely disappeared at only 36 mK. The width of the main part of transition is about 5 mK.

It can be noted that the transition temperatures during heating and cooling do not coincide with each other. This effect is due to overheating of the film, and it can be reduced by lowering the bias current.

Subsequently, the measurements of this film were repeated with several bias currents. Moreover, this film, measured after three months, changed its characteristics, and the critical temperature increased to 105 mK (Figure 4a, the red dots). It will be described in more detail below.

### 3.3. Conclusions on Measurements of the Hf-1 Films

From the measurement data for films with Hf-1, we can conclude the following.

Firstly, hafnium films thinner than 60 nm are not superconducted down to 15 mK.

The use of films of normal metals can significantly reduce the critical temperature; an example of this is the 60/20 nm Hf/Ag sample (Figure 4a).

The samples with silver layers, either at the bottom or top, demonstrate a significant change in their characteristics (marked with red dots in Figure 4a,b) with time.

The 10/75/20 nm Ag/Hf/Au film showed a more than twofold increase in the critical temperature, but at the same time retained almost the same width of the superconducting transition in the second measurement.

For 60/20 nm Hf/Ag film, the second measurement cycle also demonstrated an almost 100% increase in the transition temperature, and the slight increase in its width was also noticed.

### 3.4. Films from the Hf-2 Source

#### 3.4.1. 115 nm Hf-2 Film

A Hf-2 source for deposition was purchased from another supplier with a declared purity of 99.9%. The first investigated film with a thickness of 115 nm, deposited from this source, showed a typical IV curve with a critical current of 7.5 μA at 17 mK.

**Figure 5:**
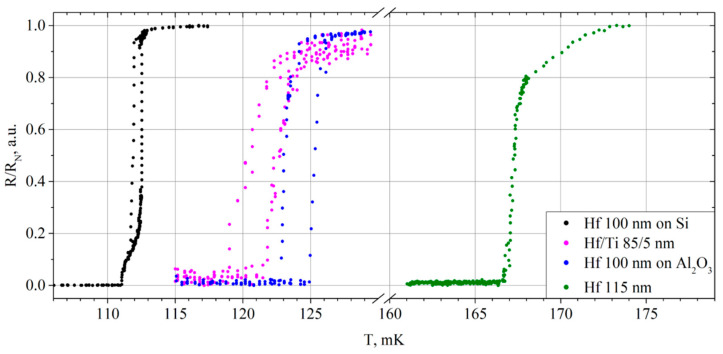
The temperature dependence of the resistance of the Hf-2 films relative to their normal resistances.

The critical temperature of the film was 166.5 mK. The transition from the superconducting to the normal state appeared as a very sharp jump, which required accurate measurements of this transition by slowly increasing the heating temperature of the plate (Figure 5, the green curve). The transition width was about 1.5 mK. Moreover, after a sharp transition, we observed a region with another slope in the temperature dependence of the resistance.

#### 3.4.2. 100 nm Hf-2 Film on Si and Sapphire and 85/5 nm Hf/Ti (Hf-2) Film on Si

In this section, we compare the properties of 100 nm hafnium films deposited on a silicon substrate with [100] orientation of thickness 480 μm and on a sapphire substrate with [111] orientation of thickness 410 μm.

The IV curves of both films were measured at 17 mK. The critical current of the film deposited on silicon was 4.4 μA, the normal resistance after the transition to the resistive state was 7.8 Ω. For the film on the sapphire substrate, these values are 3.5 μA and 7.4 Ω, respectively. It is noteworthy that the return current on the reverse branches is almost the same for both films, despite the higher critical current of the film on silicon. This confirms the expectation of better thermal conductance of sapphire, which reduces thermal hysteresis.

After measuring the IV curve, we measured the dependence of the resistance on the temperature for the bias current 1 μA. The resulting graphs are shown in Figure 5. The hafnium film on silicon showed the resistance increase at 111 mK, finishing the switching into the normal state at 113 mK. During cooling, its resistance began to decrease at 112 mK, and it turned back into the superconducting state at 111 mK (Figure 5, the black curve). The width of the main transition part was about 1 mK.

For the hafnium film on sapphire, the resistance began to increase at a temperature of 125 mK, and it completely passed to the normal state at 127 mK. Upon cooling, the transition to the superconducting state started at 125 mK, and the resistance dropped to zero at 122 mK (Figure 5, the blue curve). The thermal hysteresis between cooling and heating was slightly stronger for the film on the sapphire substrate, while in both cases a rather sharp transition is observed. The width of the main transition part was about 2 mK.

Moreover, along with these two films, an 85-nm-thick hafnium film with a 5-nm-thick titanium layer deposited on a silicon substrate was measured. In this work, the titanium layer was used to prevent the interaction of the upper hafnium surface with the atmosphere, since titanium is known for its very thin but stable oxide. The critical current of this film at 17 mK was 27 μA, and the normal resistance was about 0.2 Ω with noticeable thermal hysteresis on the IV curve despite the low resistance (Figure 2b). If we compare this film with the 97 nm Hf-1 (Figure 2a), it becomes clear that even a thin titanium layer significantly changes the film resistance and increases the critical current. It should be noted that the 5 nm titanium layer almost did not affect the critical temperature in comparison with pure Hf. The resistance of this film began to rise at 121 mK, and at 125 mK it was already in the resistive state. On cooling, the film began to lose resistance at 124 mK, and it became zero at 119 mK (Figure 5, the magenta curve). The width of the main transition part was about 3 mK.

**Figure 6:**
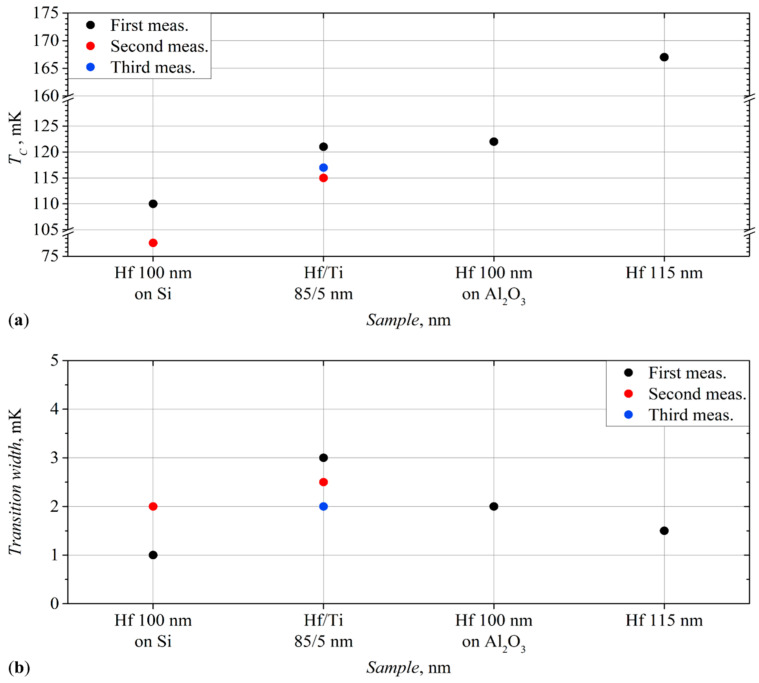
(**a**) Critical temperatures of Hf-2 films in ascending order; (**b**) Transition widths of Hf-2 films. The point size is bigger than measurement uncertainties.

All these films showed prominent results both in terms of the superconducting transition temperatures, which are close to the goal of 100 mK, and in terms of the transition width. The obtained data for these films are presented for comparison in Figure 6a,b. The temperature measurement error used for Figure 4 is also applicable to Figure 6.

Judging by the similarity of the obtained data, the fairly close critical temperatures and small transition widths are due to the characteristics of the hafnium used for deposition. At the same time, the results for the hafnium film with a thin layer of titanium on top are very close to the results for a hafnium film on a sapphire substrate. And both of these are also close to the critical temperature of the bulk hafnium [1].

It should be noted that the thinnest hafnium layer with a superconducting transition was 80 nm for the Hf-2, in contrast to 60 nm for the Hf-1. Below, we consider how the properties of films made from Hf-2 change over time.

### 3.5. Changes in Film Properties over Time

It is known that superconducting films can undergo changes in properties over time due to various factors. The most common causes of this phenomenon include:

- Oxidation. Superconducting materials are often prone to oxidation, especially at high temperatures or in the presence of oxygen. Oxidation can lead to the formation of insulating oxide layers on the surface of the thin film. Normal metals that come into contact with superconductors can also interact with atmospheric gases. For example, in air, silver creates a compound with nitrogen, forming an insulating layer with time-dependent thickness, resulting in unstable film properties [24].
- Pollution. Contamination with impurities or foreign particles can also degrade the performance of superconducting films. Even a few impurities can significantly change the superconducting properties until their complete disappearance.
- Aging and thermal cycling. Over time, superconducting films can undergo aging effects, which can lead to a gradual deterioration in their superconducting properties. In addition, repeated thermal cycles, especially between cryogenic temperatures and room temperature, can lead to thermal “fatigue” and film destruction.

To prevent a change in superconducting film properties, careful control of the conditions and processes of their production, storage, and operation is required. It is also important to develop methods for protecting superconducting films from external factors, for example, through protective coatings.

We also had to face the effects of property changes. Some films, which will be discussed below, showed unexpected and, at the same time, very essential results for detector development.

As the sample holder had 10 places, we loaded some fresh and some old films (deposited several months earlier) together. The films, therefore, experienced the same thermal cycles and were measured with the very same setup. However, to our surprise, some films displayed drastically different critical temperatures, and some did not.

#### 3.5.1. 10/75/20 nm Ag/Hf/Au (Hf-1) Film

The 10/75/20 nm Ag/Hf/Au film was measured again after 6 months. Both the critical current and the transition temperature rose to 115 μA and above 302 mK, respectively. Furthermore, the hysteresis between the direct and reverse branches of the IV characteristics increased. Most likely, this is due to the high current overheating the film. The normal film resistance has also increased, and now it is 0.4 Ω.

**Figure 7:**
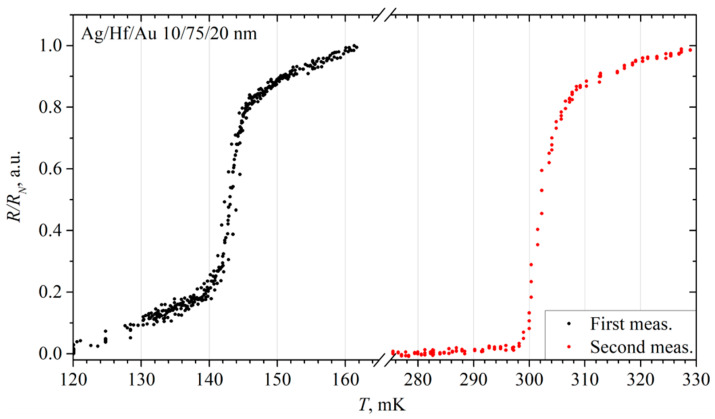
The comparative plot of the resistance versus temperature of an Ag/Hf/Au 10/75/20 nm film.

As can be seen from the comparative graph (Figure 7) of the resistance dependence on the phonon temperature, the critical temperature increased by more than twice, reaching 302 mK. At the same time, the width of the main part of the superconducting transition was almost preserved; at 306.5 mK the film had turned into its normal state. The transition width was about 6 mK for this measurement.

Such an increase in the critical temperature clearly indicates a change in the properties of the film, which can be explained by the storage time and the external factors effect.

For example, thermal cycling can cause a change in the crystal structure of the film [18], see below. The interaction with air can be excluded from possible external factors since it was covered with a golden layer.

#### 3.5.2. 60/20 nm Hf/Ag (Hf-1) Film

**Figure 8:**
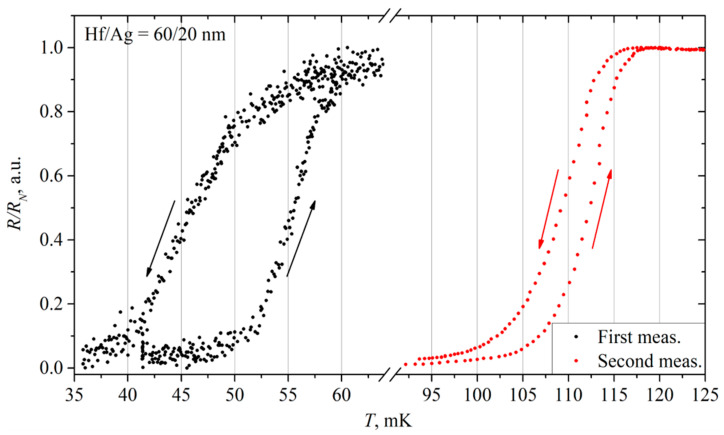
The comparative plot of the resistance versus temperature of a 60/20 nm Hf/Ag film. Arrows show the direction of resistance change during heating (up) and cooling (down).

As noted earlier, the properties of thin films can change over time. This effect was also present in the Hf/Ag 60/20 nm film, measured again after three months (Figure 8). Due to aging, the critical current increased from 21 to 41 μA. Normal resistance also increased to 0.5 Ω. At the same time, the difference between the critical and return currents was slightly reduced, meaning that the overheating of the film decreased.

As can be seen from the comparative plots of the resistance versus temperature dependence in Figure 8, the transition width remains approximately the same, but the thermal hysteresis between heating and cooling curves becomes much smaller.

The critical temperature, as for the previous sample, increased more than twice. Now, when heated, the resistance starts to increase at 105 mK, and a complete transition to the normal state is at 118 mK. When cooled, the resistance decreases from 116 to 100 mK. The transition width was 6 mK for the repeated measurement.

#### 3.5.3. 115 nm Hf-2 Film

This film has been measured several times, and the measurements clearly show the change in properties over time.

The first measurement is described above at the beginning of the previous section. In the second measurement, a week after the first measurement, the critical current dropped from 7.5 to 3.2 μA.

But at the same time, the critical temperature decreased from 166.5 mK to only 162.5 mK, and the sharp jump of resistance with a further smooth inclined increase did not go away.

#### 3.5.4. 85/5 nm Hf/Ti (Hf-2) Film

The 85/5 nm Hf/Ti film was measured for the second time after two months since the first measurement. Its IV characteristics remained completely the same, i.e., the critical current of 27 μA and the resistance of 0.2 Ω did not change, and the same hysteresis was also present due to overheating.

**Figure 9:**
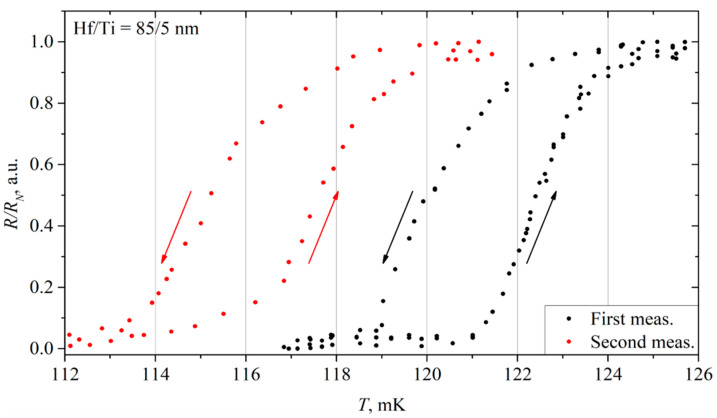
A comparative plot of the resistance of the 85/5 nm Hf/Ti film versus temperature relative to its normal resistance. Arrows show the direction of resistance change during heating (up) and cooling (down).

We measured the resistance versus temperature dependence (Figure 9). This time, the *R*(*T*) was very similar to the one measured previously. The transition was from 115 mK to 120 mK. During cooling, the resistance decreased to zero from 118 mK to 113 mK. Thus, this film almost maintained all its characteristics for two months. The transition width was 2.5 mK for the second measurement.

The changes are very minor compared to some other films’ repeated measurements. One can see that the critical temperature of the film only slightly decreased (Figure 6, the red dots), and the other properties did not change. This signals that the top layer, made of a very thin titanium film, protects hafnium from oxidation and other aging effects.

The film was measured for the third time, a month after the previous measurement. This time, the critical temperature was 117 mK (Figure 6a, the blue dots), and the transition width slightly decreased to 2 mK (Figure 6b, the blue dots). Other properties did not change, and the IV characteristics remained the same. The changes in the results compared to the two previous measurements are insignificant; we can say that the critical temperature has remained almost unchanged.

This preservation can also be explained by the peculiar properties of the titanium oxide film, which is formed on the entire surface of the sample and is very difficult to damage under any circumstances. Therefore, the investigations of hafnium films with titanium top layers will be continued. As far as we know, this combination of materials has never been used as a superconductor for sub-K superconducting devices, only as a tunnel barrier [25] for Nb Josephson junctions, so this is a novel approach for preserving the superconducting properties of hafnium or other superconducting materials susceptible to degradation.

#### 3.5.5. 100 nm Hf-2 Film on a Si Substrate

The measurements were repeated after two months for a 100-nm-thick Hf film on a silicon substrate in the same measurement cycle as the repeated measurement of the 85/5-nm-thick Hf/Ti film. 

**Figure 10:**
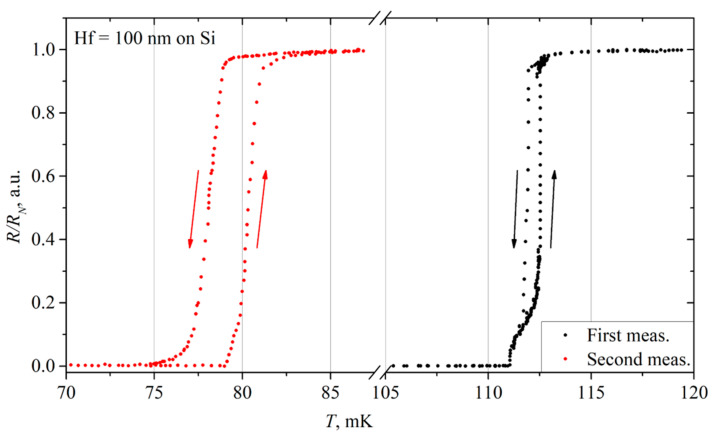
The comparative plot of the resistance versus temperature of Hf 100 nm film on Si substrate. Arrows show the direction of resistance change during heating (up) and cooling (down).

The IV curve had the same shape, but the critical current fell from 4.4 to 2.9 μA, while the normal resistance remained the same, 7.8 Ω. The *R*(*T*) is presented in Figure 10.

In this measurement, the film resistance started to increase at 78 mK, and the film fully turned into its normal state at 83 mK. During cooling, the resistance fell within the 78–75 mK range. This divergence increase between heating and cooling can be explained by higher film overheating in its normal state. The transition width was 2 mK for the second measurement.

These results differ significantly from the first tests of this film. Namely, both the critical current and the critical temperature (Figure 6, the red dots) decreased by more than 30%.

The exact reasons for such changes in these characteristics are unknown to us. It looks like the film has just degraded over time. It is yet to be determined, but we think that aging is the most likely reason.

### 3.6. Conclusions on Repeated Measurements of the Hafnium Films

The samples appear to require some form of coating that would protect the films from aging effects but would not interfere with their superconductive properties and thermal conductance. For example, as described above, the thin titanium top layer can preserve the superconducting properties of hafnium. The 85/5 nm Hf/Ti film sample in its second measurement shows very similar results to the first one, despite the time between the measurements. We attributed it to the titanium layer protecting the hafnium under it.

## 4. The Possible Reasons for Changes in the Critical Temperature of Hf Films

Our data suggest that the variation of the critical temperature of the films over time may happen due to different reasons for a single layer of Hf and for Hf with normal metal layers.

**Figure 11:**
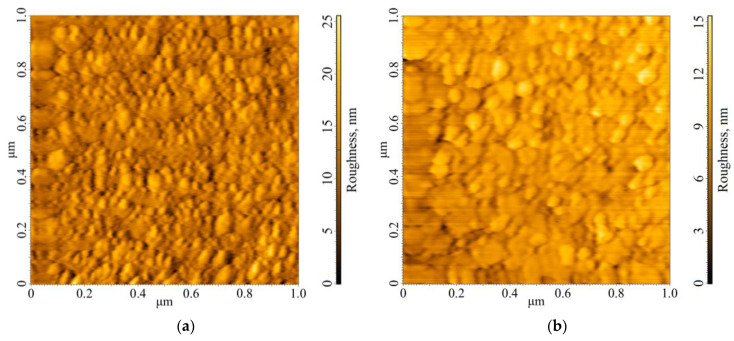
(**a**) AFM image of the Hf-2 100 nm film surface; (**b**) AFM photo of the Hf/Ag (Hf-2) 85/30 nm film surface.

Different mechanisms for changing the transition temperature also follow from AFM images taken on pure hafnium and hafnium coated with silver (Figure 11).

The film of 100 nm Hf-2 on Si [100] substrate was studied using an atomic force microscope (AFM) right after the deposition. The AFM image of the surface is shown in Figure 11a. The surface looks quite smooth, with randomly distributed grains. The average lateral size of the grains is several tens of nanometers.

Figure 11b shows an AFM image of an 85-nm-thick hafnium film covered by 30 nm of silver. In contrast to the Hf film shown in Figure 11a, this Hf/Ag film was analyzed after several measurements in the fridge. It is clearly visible that the silver coating has changed the structure of the surface; the lateral crystallite sizes are noticeably larger than in Figure 11a.

For a single hafnium, we assume that the properties may drift due to a change in the effective thickness of the hafnium film due to the growth of a HfO_2_ layer on the film surface. According to data from the Bruker diffractometer, the oxide thickness in the atmosphere grew to 2.6 nm over a month and has remained virtually unchanged since then.

As for the Hf films with layers of normal metals, there is another hypothesis. The change in the critical temperature may be associated with the recrystallization of the Hf film over time due to thermal cycling. In this case, larger crystallites can form in a film after cooling and warming. We examined several Hf/Ag (Hf-2) films with different thicknesses of silver from 10 to 30 nm on the AFM, and the surfaces look similar for all investigated silver thicknesses. It is known that silver has a great ability to diffuse into other metals. In our case, this can create defects in the hafnium crystal lattice, which in turn contributes to the formation of crystallization centers. Over a short period of time, this leads to the formation of larger crystallites, leading to relaxation of the film and changing its superconducting properties. Furthermore, this hypothesis can be confirmed by the observation, presented in [22], that hafnium films with larger crystallites have a higher critical temperature.

We also investigated the roughness of hafnium films. First, a Talysurf 2000 white light interferometer was used, and the results showed an RMS roughness of 1.67 nm in a field of 0.5 × 0.6 mm for a 60 nm film. Second, the data from an X-ray diffractometer showed a roughness of 1 nm for this film, while the actual film thickness was 57 nm. We have found that a quartz sensor in the vacuum chamber of the e-beam evaporator systematically showed smaller thicknesses of deposited hafnium films than actual values determined by Talysurf or an X-ray diffractometer. Such a difference in thickness may indicate that the film creates stress on the quartz sensor, which increases the sensor error. Therefore, we can presume that the stressed film would relax over time, including changes in its crystal structure.

## 5. Conclusions

Hafnium has very convenient properties for low-temperature applications, namely, a critical temperature close to 100 mK and a narrow width of the superconducting transition, which are required for the development of a TES detector.

We have shown that electron beam evaporation of hafnium allows obtaining superconducting films of thickness about 100 nm and roughness of just 1 to 2 nm. We have managed to decrease the critical temperature of hafnium films evaporated on Si and sapphire substrates below 100 mK, keeping the transition widths within a few millikelvins. The suppression of the critical temperature is realized by the proximity effect with layers of normal metals such as silver and gold.

We have observed the instability of the transition temperatures in some films due to aging effects. In single-layer Hf films, the critical temperature tends to decrease with time, while in bi- and three-layer samples with normal metals, the transition temperature significantly increases. In the latter case, the critical temperature for some films increased twofold since the first measurement. It can be explained by Hf recrystallization caused by silver after thermal cycling.

We have proposed and tested a solution for how to preserve the superconducting properties of hafnium films from aging. For this, we have applied the novel approach of using thin titanium top layers with a thickness of about 5 nm. It has not previously been used with hafnium for superconducting applications at such low temperatures. The layer with this thickness is thick enough to keep the critical temperature and critical current of the film stable over time and after thermal cycling. The combination of hafnium and titanium films appears to be promising for further development of a TES microcalorimeter based on hafnium films.

## Figures and Tables

**Table 1:** The resistivity of hafnium films.

Sample	Thickness, nm	ρ (300 K), Ω × m	ρ (20 mK), Ω × m
Hf-1 on Si	97	9.7 × 10^−7^	5.3 × 10^−8^
Hf-1 on Si	60	6.7 × 10^−7^	5.4 × 10^−8^
Hf-2 on Si	115	6.9 × 10^−7^	3.5 × 10^−7^
Hf-2 on Si	100	3.3 × 10^−6^	3.9 × 10^−7^
Hf-2 on Al_2_O_3_	100	1.2 × 10^−5^	3.7 × 10^−7^

## Data Availability

The data that support the findings of this work are available from the corresponding author upon reasonable request.
